# Commercial applications of quantum computing

**DOI:** 10.1140/epjqt/s40507-021-00091-1

**Published:** 2021-01-29

**Authors:** Francesco Bova, Avi Goldfarb, Roger G. Melko

**Affiliations:** 1 Rotman School of Management, University of Toronto, Toronto, Canada; 2 Creative Destruction Lab, Toronto, Canada; 3 Dept. of Physics & Astronomy, University of Waterloo, Waterloo, Canada; 4 Perimeter Institute for Theoretical Physics, Waterloo, Canada

## Abstract

Despite the scientific and engineering challenges facing the development of quantum computers, considerable progress is being made toward applying the technology to commercial applications. In this article, we discuss the solutions that some companies are already building using quantum hardware. Framing these as examples of combinatorics problems, we illustrate their application in four industry verticals: cybersecurity, materials and pharmaceuticals, banking and finance, and advanced manufacturing. While quantum computers are not yet available at the scale needed to solve all of these combinatorics problems, we identify three types of near-term opportunities resulting from advances in quantum computing: quantum-safe encryption, material and drug discovery, and quantum-inspired algorithms.

## Introduction

Computers do arithmetic. Underlying all the amazing applications of computers today is arithmetic, calculated using binary digits or “bits.” The UNIVAC computer of the early 1950s could perform about 465 multiplications per second, much faster than the “human computers” who performed such calculations for the military and other organizations, as highlighted in the movie *Hidden Figures*. Today’s computers are billions of times faster.^1^ However, despite these advances, there is an important class of arithmetic problems that remain out of reach for classical computers for the foreseeable future: Large-scale, combinatorics calculations. Combinatorics problems involve the way in which items are arranged. As the number of items grows, the number of possible permutations grows exponentially. The typical objective in combinatorics calculations is to find a specific value, and this exponential growth in the number of permutations makes it increasingly challenging to find that specific value.

Understanding technological change as a drop in the cost of some factor has a long history in the economics and management literatures, for example arithmetic for computers, search for the internet, and prediction for machine learning.^2^ In this paper, we argue that—from a management perspective—quantum computing improves combinatorics calculations. We then provide a number of examples of industries in which better combinatorics calculations may be transformative.

To start, it is important to note that a surprising number of practical problems can be viewed as combinatorics problems, including applications in cryptography, chemistry, materials science, finance, and advanced manufacturing. Many of these applications have remained out of reach for classical computers because the number of possible permutations often grows to a point that they could take thousands or millions of years to assess, if each possibility was assessed sequentially.

Combinatorics problems expose the limits of classical computers, and therefore represent the potential for quantum computers. We are not there yet however. Quantum computers do not work at the required scale, and there are major technical issues that remain in the race to scale up today’s quantum hardware. Despite these limitations, considerable progress is being made toward applying present-day quantum hardware to commercial applications. Below, we provide examples of combinatorics problems that companies are already working to solve, driven by advances in quantum computing. We divide these applications into four industry verticals: cybersecurity, materials and pharmaceuticals, banking and finance, and advanced manufacturing. Despite the remaining technical challenges in developing large scale and practical quantum computers, we identify three types of near-term opportunities generated by advances in quantum computing.

First, the development of algorithms for quantum computers has led to advances in our ability to solve large-scale combinatorics problems on already-available classical computers. We have seen a large number of quantum companies, via their work with emerging quantum computing technology, develop “quantum-inspired” algorithms that enable them to solve important practical problems on classical computers.^3^ As new algorithms are discovered, the boundary between quantum and classical can shift. Quantum algorithms can inspire insight into new classical solutions. In other words, the rise of quantum-inspired solutions suggests that quantum computing could change the speed of technological advancement for combinatorics problems, even if quantum computers never achieve the scale and reliability needed to dominate other types of computing.

It is not unusual for an emerging technology to push innovation in existing technologies, without displacing the incumbent. This was the case in the TV market, where innovation in LCD screens accelerated after the emergence of commercially viable plasma flat screen televisions. Eventually, innovation in LCD screens outpaced innovation in plasma, so that the emerging technology never really displaced the incumbent.^4^

However, it is unclear whether a similar phenomenon will play out in quantum, particularly since many different models and architectures are still being explored. Current quantum devices are small and noisy, while the holy grail for the technology is to achieve large, highly-controlled, coherent, analog or digital quantum computers.^5^ In short, a computer where the rate of component failure is sufficiently low to deliver uninterrupted service. Even for the most complex combinatorics problems, it could be that quantum-inspired classical computers ultimately dominate, or that one or several quantum computer designs ultimately dominate, or that some kind of hybrid approach yields a market leader. For now, however, we know that quantum algorithms have inspired useful innovations in software for classical computers that have generated commercial opportunities.^6^

Second, the threat of the arrival of quantum computers in the near future suggests benefits to investing in certain technologies today—most notably, in cryptography.^7^ If a fully functioning sufficiently coherent quantum computer becomes available, many files encrypted using current standards would be more easily decipherable. Therefore, if something needs to remain encrypted for many years, the threat that a quantum computer may be available in a decade or two means that it is worthwhile investing in quantum-safe encryption today.

Third, quantum computers were originally conceived by Richard Feynman to simulate or emulate quantum systems that are difficult to simulate classically on a classical computer.^8^ As molecules grow more complex, with more atoms and more electrons in those atoms, the number of possible configurations grows exponentially. This type of chemical engineering requires combinatorics, and so is often not well-suited to classical computers. In other words, materials development and drug discovery are constrained by the combinatorics challenges that arise from simulating new molecules and new applications of molecules. Quantum simulators, which are attainable in near-term devices, should help. While we are still waiting for an example of a new useful molecule that has been discovered through simulation on a quantum computer, complex known molecules have been simulated, demonstrating proof-of-concept.

Both the short-run and long-run opportunities with quantum computing will depend on the particular quantum computing architectures developed. There are many such architectures, each with their strengths, weaknesses, and corresponding technical challenges. This management-focused article will not provide a technical overview. Just as most articles about the impact of other types of information technology on business don’t detail the physics of semiconductors or the underlying mathematics of a Turing machine, we will not detail what quantum computing is, how it works, and how the various architectures differ.^9^ Our purpose is then to use examples of companies working with quantum technology to shed light on the types of problems for which these advancements are likely to be most useful, in the near-term and in the long run.

## Quantum and combinatorics

As the name suggests, combinatorics problems ask the question “how many ways can this set of objects be combined?”.^10^ Such problems can also ask whether a certain combination is possible, or what combinations of objects are “best” by some metric. In many cases the number of tasks required to answer these questions—such as counting all possible combinations—grows exponentially as the number of objects grows. This makes finding the answer computationally challenging.

The promise of quantum computers lies in their potential to drastically reduce the time it takes to solve these sorts of problems by utilizing algorithms that make use of quantum effects. Not all combinatorics problems require quantum computers. There are combinatorics problems that are comparatively easy for humans as well as classical computers and sufficiently large and coherent quantum computers to solve (i.e., trying every sequence of 2^2^, 2^3^, and 2^4^ combinations). There are combinatorics problems that are challenging for humans to solve, but easy for classical computers as well as for sufficiently large and coherent quantum computers to solve (i.e., trying every combination on a gym lock). Notably there is no benefit to having a quantum computer solve either of these sorts of problems, because we can work through these problems with our existing classical computers, and these classical computers suffer comparatively fewer shortcomings compared to quantum computers.

Next there is a class of combinatorics problems which are challenging for classical computers to solve, but may be comparatively easy for a sufficiently large and coherent quantum computers to solve. These are the types of problems where quantum computing could prove useful, and therefore these are the types of problems that are the focus of our discussion.

Finally, there are a class of combinatorics problems that may be too challenging for even a sufficiently large and coherent quantum computer to process in a timely manner. These types of problems, while not our focus, are still important to understand, because they define boundaries that even quantum computing cannot solve.

Currently, quantum computers are not large or reliable enough to solve any practical problems better than classical computers, although Google has recently demonstrated quantum “supremacy” on a complex problem of little practical use.^11^ As laid bare in this study, there are many fundamental and technical challenges still facing quantum hardware manufacturers. If and when sufficiently large and reliable quantum computers do become available, they will be able to conduct a wide variety of calculations, with their core advantage being in some areas that classical computers will struggle with, such as large-scale combinatorics problems.

## Cybersecurity

Perhaps more than any other application, the use of quantum technology in cybersecurity has pushed government attention and public sector investment. The World Economic Forum has highlighted that “quantum computing could make today’s cybersecurity obsolete.”^12^ When the US passed the Quantum Initiative Act in 2018 to fund quantum computing, cybersecurity issues were at the center of the discussion.^13^ Quantum computing has also received attention in the context of great-power competition between the US and China, again with an emphasis on security generally, and cryptography in particular.^14^

Underlying this attention and investment is the recognition that the technology underlying modern cryptography uses combinatorics.^15^ In 1994, Peter Shor showed that certain types of encryption would become substantially less complex to break with a sufficiently large and coherent quantum computer.^16^ The most notable technical point is that the RSA public-key standard for asymmetric encryption would be reduced from an exponential complexity to a polynomial one. In other words, a widely used encryption standard would be compromised. Thus, while quantum algorithms apply to many combinatorics calculations, there is a clear and direct application to cryptography. Shor’s algorithm in particular highlights the opportunity for solving large-scale problems (or creating a large-scale threat) with fully functional and reliable quantum computers. That does not mean that current cybersecurity solutions will be broken soon. Sufficiently reliable, large-scale coherent quantum computers do not exist and the timeline for their arrival is unknown.

Nevertheless, cybersecurity does represent a near-term opportunity for those who understand quantum computing. Key exchange for encrypted transmission is the main threat of Shor’s algorithm, meaning that communications will be vulnerable if and when large-scale quantum computers appear. However, there are business opportunities today in preparing for a future of quantum decryption. In some contexts, long-term encryption of files is important, such as in the United States where classified information must remain secure for decades.^17^ The security of stored data is threatened in particular when the key exchange data used to encrypt a file is vulnerable to interception. Then, even a small probability of a large scale quantum computer creates a commercial opportunity in quantum-safe encryption.^18^ Just like fear of a possible Y2K bug led to massive investment in computer system upgrades, fear of a possible quantum computer using Shor’s algorithm means that developing quantum-safe encryption systems will be prudent in specific sectors. Note, this is where also understanding the limitations of quantum computers is critical. The potential of quantum computing should lead to a change in cryptography practices such as longer key lengths, but not an end to encryption. To this end, the National Institute of Standards and Technology (NIST) is in the process of selecting public-key cryptographic algorithms that are “capable of protecting sensitive information well into the foreseeable future, including after the advent of quantum computers.”^19^

There is a small but growing industry focused on helping companies prepare for the potential end of the usefulness of current encryption techniques.^20^ These companies focus both on hardware, such as quantum key distribution, and software solutions. For example, KETS Quantum Security, a company developing quantum safe solutions, is developing thumbnail-sized, on-chip quantum encryption hardware.^21^ Additionally, evolutionQ helps organizations “Prepare for the Quantum Age.” Founded by University of Waterloo professors and quantum computing/cryptography experts Michele Mosca and Norbert Lütkenhaus, evolutionQ provides quantum risk assessment, risk management, and cybersecurity solutions.^22^ In marketing their products, evolutionQ emphasizes the uncertainty in the timing of the arrival of quantum computers. Their tools do not require quantum computers to work. They are focused on preparing systems for the potential for quantum computers to hack existing encryption techniques. In other words, while the expertise of the company founders is in quantum computing, their near-term solutions are largely classical.

## Chemical engineering

Another area where quantum holds promise is in material discovery and drug development. As noted above, the original conception of a quantum computer—by Nobel Prize winner Richard Feynman in a 1982 paper—was as a tool for simulating quantum processes, such as the workings of a collection of atoms.^23^ Subsequent applications related to the potential for a quantum advantage when solving certain combinatorics problems did not become clear until Shor discovered his algorithm in 1994.

Developing new useful molecules requires combinatorics because there are many possible combinations of atoms, and many possible ways that they can bond. The histories of material discovery and drug development are full of stories that discuss the impact of serendipity and luck on discovery.^24^ For example, Ivermectin is a drug that was first used as a treatment for heartworm in animals. In the 1980s, the drug was further developed as an effective treatment for onchocerciasis (also known as river blindness) and has gone on to improve the quality of life for hundreds of millions of people. Nearly as remarkable as the drug’s impact on the world, is the amount of luck that needed to occur for the discovery to be made in the first place.^25^ In the 1970s, Satoshi Ōmura, a scientist from the Kitasato Institute in Japan, took a sabbatical in the United States. While there, he struck up a partnership with Merck. Upon returning to Japan he took soil samples and sent interesting bacteria from those samples to the U.S. for Merck to assess. One of these samples, taken near a golf course in Japan, contained bacteria which eventually was used to develop Ivermectin.

In recent years, scientists have increasingly turned to computational chemistry simulations for these applications. With computational chemistry, scientists try to understand *both* a material’s molecular structure and its properties. Based on these simulations, scientists then choose the best candidates to ultimately synthesize. The goal of computational chemistry has led to cost efficiencies in the R&D process relative to prior methods which involved trial and error or simply, luck.

However, computational chemistry simulations are in and of themselves challenging. First, a molecule‘s properties are strongly influenced by its lowest energy state. Thus, to generate inferences on both a molecule’s structures and its expected chemical properties, the starting point of many simulations is to identify the structure that will yield a molecule’s lowest energy state. Here, combinatorics problems again arise. For example, simulating a molecule involves assessing the interaction between every electron and every proton in every atom in the molecule and the interaction of those particles with every other atom in the molecule. Notably every electron from every atom is repelled from every other electron from every other atom, and every electron from every atom is attracted to every proton from every other atom. Thus, the addition of an incremental atom to a molecule can lead to an exponential increase in the number of interactions that have to be accounted for, as the electrons and protons of the new atom will be interacting with those from existing atoms in the molecule.

Because of this combinatorics challenge, only comparatively small molecules are accessible to highly-accurate, generic solutions using classical computers.^26^ Many computationally cheaper approximations are available, and in some cases provide sufficiently accurate results, but in many other cases are known to fail. In this space, some combinatorics problems can be formulated such that even a noisy quantum device may provide an advantage.^27^ One approach, pioneered by D-Wave Systems over the last 20 years, is to explore optimization problems that might display an advantage for an algorithm called “quantum annealing.” Quantum annealing aims to find the best solution to a problem by exploiting the tendency of quantum mechanics to “tunnel” through barriers between different possible solutions. In September 2020, D-Wave launched a new hardware system with 5000 qubits, useful for this purpose. Since the approach may be amenable even on relatively noisy quantum devices, it has inspired other hardware manufacturers to explore a variety of algorithms for their own current hardware. Optimism regarding quantum computing’s eventual impact on chemical engineering is high, in particular following IBM Q’s simulation of beryllium hydride in 2017. As University of Toronto scientist and quantum entrepreneur Alan Aspuru-Guzik suggested following IBM Q’s achievement “When quantum computers are able to carry out chemical simulations in a numerically exact way… This may lead to the discovery of new small-molecule drugs or organic materials.”^28^ The implication is that there are classes of molecules that are too challenging to simulate with classical methods because of the underlying combinatorics, but will be possible to simulate in a timely manner once quantum computers improve sufficiently. If and when this point happens, then this will lead to further improvements in R&D cost efficiency over current computational methods.

Even without a full-scale quantum computer, quantum methods already show some promise. For example, OTI Lumionics is one of many companies that uses a computational approach to molecule discovery in which they try to determine a molecule’s structures and properties jointly. Again, the intent when simulating a molecule is to model the interaction between all of its electrons, and these aspects of the process that pose combinatorics obstacles. OTI starts with thousands of potential candidates to simulate and then pares the list down using computational methods to those that may have the desired properties based on estimates of molecular structure.^29^

OTI is experimenting with different quantum hardware platforms for materials creation. They are one of the first companies to model their problems in a way that can be implemented on D-Wave’s quantum annealing hardware.^30^ Their work in quantum has provided a two-fold benefit for the firm, one long term and one near term.^31^ The long-term benefit is that OTI will be quantum ready if and when quantum computers become sufficiently powerful and noise has been sufficiently reduced.

The short-term benefit is that OTI’s work in quantum has yielded near-term returns by helping the company to derive quantum-inspired algorithms that they can then apply on current classical computing architectures. Modelling their problem for the D-Wave machine directly led to quantum-inspired algorithms that provided efficiency improvements relative to previously existing classical algorithms. Recently, OTI was one of four companies to be invited to use Microsoft’s new QIO quantum-inspired service as part of Azure Quantum, where new evidence suggests that OTI’s quantum-inspired algorithms combined with Azure Quantum on classical hardware perform better than other classical methods that can be run on the same hardware.^32^ These improvements on classical hardware are important. According to OTI’s CEO Michael Helander, the cost to experimenting with quantum (measured as the opportunity cost to assigning staff to work with quantum technologies as opposed to some other platform) has been outweighed, even in the near term, by the benefit derived from creating these new quantum-inspired algorithms.

Several other companies are exploring the use of quantum computing for material or drug discovery. For example, Menten AI utilizes a combination of quantum computing, synthetic biology, and machine learning to aid in the creation of new proteins.^33^ Another example is Zapata Computing, who pioneered a number of near-term quantum computing methods for chemical simulations. Zapata has developed a commercial software platform featuring quantum libraries with applications in chemistry, biopharma, machine learning, and more.^34^

## Banking / Finance

Combinatorics challenges are common in banking and finance, from arbitrage to credit scoring to derivatives development. One way banks and other financial institutions deal with these problems is to constrain them in order to make them more tractable. In other words, banks simplify the problems to reduce the set of possible solutions. Constraining the set of possible solutions means that sometimes the best solution is never found. There is a potential for quantum computers to shed insights into larger problems where constraints are relaxed and where more outcomes are possible.

Many of these challenges relate to the classic “traveling salesman problem” that has been a staple of operations research for decades.^35^ The idea of the traveling salesman problem is that one salesman has a number of cities to travel to and needs to travel to every city once. The goal is to find the shortest route that: (1) goes to each city once, and (2) ends up in the starting city. From a value proposition perspective, the benefit to using the shortest route is straightforward: The salesman will presumably generate the same revenue by travelling to each city, while minimizing travel costs by pursuing the most efficient route. It is relatively straightforward to find the shortest route when there are comparatively few cities to travel to (e.g., four). However, the problem becomes less and less tractable the more and more cities that are added. When the number of cities that need to be travelled to gets sufficiently large, quantum computing may eventually offer the potential to speed up the process to the point that finding a global minimum “route” through all possible cities becomes possible. A surprisingly large number of business problems can be framed as variations of the traveling salesman including circuit design, package delivery, and train scheduling. More specifically, researchers have identified combinatorics problems in banking and finance that might benefit from quantum computing, including portfolio optimization, foreign exchange arbitrage, and credit scoring.^36^

In credit scoring, for example, banks use data to predict which customers are likely to default, and which customers are likely to repay their loans. There are two types of errors that banks might make when making a loan decision. One type of error arises when a bank’s credit scoring model suggests lending money to a client and then the client subsequently defaults. Another type of error arises when a bank’s model suggests not lending a customer money but the customer would not have defaulted had the bank given the loan. It is costly to lend money to people who default. It is also costly to refuse profitable customers.

With the preceding as a backdrop, one might assume that banks would like to incorporate as many different factors as possible when credit scoring. However, a paper by quantum computing software company 1Qbit highlights a cost to using a large number of factors: verifying the accuracy of the information.^37^ After all, without robust verification, borrowers may omit key information or outright lie. Therefore, lenders might be willing to sacrifice prediction accuracy for a reduced cost of verifying the accuracy of a loan application. Using data from lending decisions and relevant customer characteristics, the paper demonstrates the combinatorics challenges of determining which information to collect in order to generate accurate predictions without spending too much on verifying the accuracy of the data. These are combinatorics problems because every possible grouping of customer characteristics needs to be assessed. So, if there are one hundred possible borrower characteristics, factors 1, 3, and 15 need to be compared to factors 2, 9, 22, 51, and 85 and so on. Importantly, the number of possible combinations to assess increases exponentially with every additional factor.

Many other financial problems involve understanding the set of possible outcomes for a number of assets. For example, the decision to invest in a portfolio of stocks involves simulating the distribution of possible future prices from the portfolio’s underlying stocks. With a small number of underlying assets to model, these simulations are relatively straightforward, and banks and financial institutions use a tool called Monte Carlo simulation. These simulations are widely used for derivatives pricing and risk management.^38^

As the number of underlying assets and factors grows, the pricing of advanced derivatives and the construction of value-at-risk models can require simulation of the joint distribution of a large number of assets. These are combinatorics problems because the future value of one asset may be related to the values of the other assets. Risk assessment requires more than knowing the possible set of future values of the various assets, it involves knowing how those values relate to each other. For example, suppose a bank wants to conduct a risk assessment on a mortgage portfolio for homes in Florida and Nevada. If real estate prices in those states move together, so that a crash in Florida prices means a likely crash in Nevada prices, then that portfolio is likely to be risky. In contrast, if the prices are independent and do not move together, then the risk of that portfolio is lower. If we add all other US states, plus many other countries, and non-mortgage assets, the complexity of this problem increases substantially.

In such a setting, Monte Carlo becomes very slow on a classical computer and this limits the ability to price complex derivatives or simulate value-at-risk models in a timely manner. Interestingly, a McKinsey report notes that many banks have reduced the use of Monte Carlo simulations for value-at-risk calculations. The report cites increasing computational complexity as a possible reason, emphasizing that the number of factors that banks need to simulate has grown over time.^39^ The potential for quantum-accelerated Monte Carlo simulation is a speed-up with respect to the underlying simulation itself,^40^ and this may enable new types of derivatives to be priced or risk models to be simulated more quickly.

Given the computational intensity of many banking and finance problems, there is potential for profitable applications of quantum computing as the technology matures. Some companies have already made progress. For example, Cogniframe is developing a “Financial Services Operating Layer” that will be positioned atop the Quantum Cloud and will be used to help solve challenging optimization and simulation problems.^41^ In addition, Multiverse Computing is using quantum and quantum-inspired algorithms to develop a comprehensive software suite to solve quantitative financial and macro-economic simulation problems.^42^ Such applications are promising and some may not require a fully functioning large scale general purpose quantum computer to provide business value. Furthermore, this is another area where quantum-inspired algorithms might arise that make it easier to solve combinatorics problems on classical computers.

Overall, combinatorics problems are common in finance and banking, and solving them would be extremely valuable. Nevertheless, the highest value combinatorics problems in finance and banking will likely require substantial advances in quantum computing technology. Until such technology becomes available, there is near-term potential for quantum-inspired algorithms to generate profit opportunities.

## Advanced manufacturing

Solid State AI is working to make advanced manufacturing more efficient. They are focused on helping companies identify the cause of rare failures in their manufacturing processes. Failure, although rare, can be very costly and so predicting these events can generate enormous savings. These are difficult combinatorics problems, related to finding a single fault in systems where many possible sequences need to be investigated.

This is an issue that often arises with unbalanced datasets. Most of the time, the processes operate as expected, and failures are rare. This means there is not much data on past failures relative to the number of things that could go wrong. This also means that it is challenging for standard statistical analyses to explain failure rates, as many possible paths could explain the same failure. Assessing why processes fail in this setting is made even more complicated when there are many possible combinations of events that have to be assessed to understand why failure occurred—i.e., when the signal-to-noise ratio is very low. The problem then is similar to the travelling salesman problem mentioned above, where the goal is to find the shortest route among many possible routes. Here, the goal is to find the sequence of events that led to failure among many possible sequences.^43^

In many advanced manufacturing processes, failure is rare but very costly. For example, in the process of making CPU chips, there may be 2 failures in 10,000 runs once the machines are properly calibrated. Additionally, there can be thousands of steps in the process to manufacture a chip, and every step in the process may have different sensors and indicators that can take on different readings.

In this setting, the number of combinations that need to be assessed increases exponentially with each new process step (and every set of new indicators that accompany that process step). If failure is common (e.g., 20% failure rate), then conventional statistical methods to inferring causal links between various factors and failure would become viable. Furthermore, for less advanced manufacturing, like clothing manufacturing which can involve as few as 15 steps, standard solutions are also more feasible because the combinatorics problems aren’t as challenging. Put differently, although solving the problems gets more challenging with each step, there are materially fewer steps in this process, and thus far fewer combinations to assess. Thus, quantum computing is not as likely to provide a benefit beyond current methods in settings with high failure rates or in less advanced manufacturing with fewer steps.

Solid State AI assesses many challenges in advanced manufacturing, including process failure when failure is rare. Solid State AI has been experimenting with quantum hardware providers to understand and reduce such failures.^44^ While the hardware needed for a quantum solution is not yet reliably available at scale, Solid State AI has been able to generate value for advanced manufacturers through the development of quantum-inspired classical algorithms that provide improved solutions using classical computing infrastructure.

There are two aspects of the value proposition for advanced manufacturers to utilizing these sorts of quantum-inspired solutions to enable better prediction of rare but costly failures. We can illustrate both benefits with DuPont Analysis.^45^ In one specification of the formula, DuPont Analysis involves the disaggregation of return on assets (ROA) into two different ratios: profit margin and total asset turnover.^46^ The profit margin is often an indicator of the nature of competition in the market or the price setting/price taking nature of the firm or its industry. Total asset turnover measures the firm’s efficient use of its assets.

For most companies, the two ratios move in opposite directions. For example, a dollar store will typically have lower margins, as it competes on price, but higher turnover because its products are affordable to a broader number of consumers. Advanced manufacturers like chip makers may have lower asset turnover, because it takes a long time to manufacture a chip, but presumably higher margins than a dollar store because not every company has the technical expertise to enter the chip market (and profit margins are often increasing in the barriers to entering a market).

Making better predictions with unbalanced data through quantum-inspired solutions might improve ROA in two ways. First, by reducing downtime, efficiency and (in turn) asset turnover should improve. Improved total asset turnover should have a positive effect on ROA. Second, the firm becomes more cost efficient which should improve profit margins. Improved profit margins should also improve ROA.

## Conclusion

Billions of dollars are now pouring into quantum computing.^47^ We have argued that the biggest promise of such computers lies in solving large combinatorics problems. Quantum is poised to make solving large scale combinatorics problems faster and cheaper. The examples highlighted above in cybersecurity, chemical engineering, banking and finance, and advanced manufacturing suggest that these problems are common and occur in a wide range of industries. In order to assess where opportunities lie in any industry, the first step is to identify combinatorics problems that could generate substantial value if unlocked.

We have also emphasized that quantum computers are not yet available at the level of power and reliability needed for solving these types of problems. Still, we highlighted three near-term commercial opportunities from recent advances in quantum hardware and software: (1) quantum-safe encryption on classical computers, (2) material and drug discovery, and (3) new quantum-inspired algorithms for classical computers.

Before concluding, it is important to recognize that the set of potential applications highlighted above does not represent the full set of business opportunities that leverage recent advances in engineering related to quantum phenomena. In particular, quantum sensing and quantum communication are emerging technologies with commercial potential.^48^ We have also not discussed companies focused on building the quantum ecosystem, including quantum computing companies like Agnostiq, which provides additional encryption for users who work on quantum-based computers via the cloud.^49^ Finally, we have also not discussed companies who are working on developing new types of quantum computers. Our focus in this article has been on the application of quantum computing for business and management problems that are challenging because of the underlying combinatorics.

There is much we do not know. The quantum machines that are likely to be available (even in the near future) may prove useful for applications that remain undiscovered. As University of Toronto scientist and quantum entrepreneur Alan Aspuru-Guzik put it in an interview with *Nature* magazine, there is “a role for imagination, intuition and adventure. Maybe it’s not how many qubits we have; maybe it’s about how many hackers we have.”^50^ For now, given current insights, if quantum computers prove useful, it will be to businesses and organizations that identify high value combinatorics problems that cannot be solved well on classical computers.

## Data Availability

Not applicable.

## Footnotes

1 P. Ceruzzi, “A History of Modern Computing,” 2nd ed. (Cambridge MA: The MIT Press, 2003).

2 W. Nordhaus, “Two Centuries of Productivity Growth in Computing,” Journal of Economic History; volume 67, no. 5 (March 2007): 128–159. C. Shapiro and H. Varian, “Information Rules,” (Boston MA: Harvard Business School Press, 1998). A. Agrawal, J. Gans, and A. Goldfarb, “Prediction Machines: The Simple Economics of Artificial Intelligence,” (Boston MA: Harvard Business Review Press, 2018).

3 J. Miguel Arrazola, A. Delgado, B. Roy Bardhan and S. Lloyd, “Quantum-inspired algorithms in practice,” arXiv:1905.10415, 2019.

4 https://www.techwalla.com/articles/the-history-of-flat-screen-tvs

5 J. Preskill, “Quantum Computing in the NISQ era and beyond,” Quantum 2, 79, 2018.

6 S. Mugel, C. Kuchkovsky, E. Sanchez, S. Fernandez-Lorenzo, J. Luis-Hita, E. Lizaso and R. Orus, “Dynamic Portfolio Optimization with Real Datasets Using Quantum Processors and Quantum-Inspired Tensor Networks,” arXiv:2007.00017, 2020.

7 N. Gisin, G. Ribordy, W. Tittel, and H. Zbinden, “Quantum cryptography,” Rev. Mod. Phys. 74, 145, 2002.

8 Richard Feynmann, “Simulating Physics with Computers,” International Journal of Theoretical Physics, 21, 467, 1982.

9 We refer the interested reader to National Academies of Sciences (NAS), Engineering, and Medicine, “Quantum Computing: Progress and Prospects” (Washington, DC: The National Academies Press, 2019). For a more business-oriented introduction see A. Menard, I. Ostojic, M. Patel, and D. Volz, “A Game Plan for Quantum Computing,” February 6, 2020, see https://www.mckinsey.com/business-functions/mckinsey-digital/our-insights/a-game-plan-for-quantum-computing?cid=other-eml-alt-mcq-mck&hlkid=1fe54a0e34b94bb9a66bb2f8aef7d592&hctky=1438625&hdpid=a13cffc2-b5b9-4731-ab95-160393a12b79.

10 D. Guichard, “An Introduction to Combinatorics and Graph Theory” (https://www.whitman.edu/mathematics/cgt_online/cgt.pdf).

11 F. Arute *et al*. “Quantum supremacy using a programmable superconducting processor,” Nature 574, 505 (2019).

12 P.H. Adams, “Why Quantum Computing Could Make Today’s Cybersecurity Obsolete,” July 26, 2019, https://www.weforum.org/agenda/2019/07/why-quantum-computing-could-make-todays-cybersecurity-obsolete/.

13 C. Monroe, M.G. Raymer, J. Taylor, “The U.S. National Quantum Initiative: From Act to Action,” Science 364, no. 6439 (2019): 440–442; Authenticated U.S. Government Information (GPO), “National Quantum Initiative Act” (Washington D.C.: Government Printing Office, 2018); A. Herman, “At Last America is Moving on Quantum,” August 20, 2018, https://www.forbes.com/sites/arthurherman/2018/08/20/at-last-america-is-moving-on-quantum/#71eaa5d55327; National Science and Technology Council, “National Strategic Overview for Quantum Information Science” (Washington D.C.: 2018).

14 E.B. Kania and J. Costello, “Quantum Hegemony? China’s Ambitions and the Challenge to U.S. Innovation Leadership,” September 12, 2018, https://www.cnas.org/publications/reports/quantum-hegemony; E.B. Kania, “China’s Quantum Future: Xi’s Quest to Build a High-Tech Superpower,” September 26, 2018, https://www.foreignaffairs.com/articles/china/2018-09-26/chinas-quantum-future.

15 E.g. C. Paar and J. Pelzi, “Understanding Cryptography: A Textbook for Students and Practitioners” (New York: Springer, 2009).

16 P.W. Shor, “Polynomial-Time Algorithms for Prime Factorization and Discrete Logarithms on a Quantum Computer” *SIAM Journal of Computing* **26**, 1484 (1997).

17 E.g. Office of the Press Secretary, “Executive Order 12958” (Washington D.C.: 1995).

18 M. Mosca, “Cybersecurity in an Era with Quantum Computers,” IEEE Security Privacy; volume 16, no. 5 (September 2018): 38–41.

19 Status Report on the Second Round of the NIST Post-Quantum Cryptography Standardization Process (NISTIR 8309), https://doi.org/10.6028/NIST.IR.8309.

20 T. Simonite, “Quantum Computing Paranoia Creates a New Industry,” MIT Technology Review, January 2017, https://www.technologyreview.com/s/603424/quantum-computing-paranoia-creates-a-new-industry/.

21 https://kets-quantum.com

22 www.evolutionq.com

23 R.P. Feynman, “Simulating Physics with Computers,” International Journal of Theoretical Physics 21 (June 1982): 467–488; G. Popkin, “Waiting for the Quantum Simulation Revolution,” American Physical Society Physics 12, no. 112 (October 2019).

24 T. Ban, “The role of serendipity in drug discovery.” *Dialogues in Clinical Neuroscience* 8, 335 (2006).

25 S. Rovner, “Discovery of Ivermectin,” American Chemical Society (2016).

26 See, for example, Popkin (2019), above footnote 13.

27 Y. Cao *et al*., “Quantum Chemistry in the Age of Quantum Computing,” *Chem. Rev.* 2019, 119, 19, 10856 (2019).

28 “IBM Pioneers New Approach to Simulate Chemistry with Quantum Computing,” IBM News Room, Wednesday, Sept. 13, 2017.

29 A. Izmaylov, T.-C. Yenab and I. Ryabinkin, “Revising the measurement process in the variational quantum eigensolver: is it possible to reduce the number of separately measured operators?”, Chem. Sci., 10, 3746 (2019); I. Ryabinkin and S. Genin, “Symmetry adaptation in quantum chemistry calculations on a quantum computer,” arXiv:1812.09812 (2018).

30 S. Genin, I. Ryabinkin, A. Izmaylov, “Quantum chemistry on quantum annealers,” arXiv:1901.04715 (2019).

31 These insights were generated from discussion with Michael Helander, President and CEO, OTI Lumionics.

32 Microsoft Quantum Team, “OTI Lumionics: Accelerating Materials Design with Azure Quantum,” January 21, 2020, https://cloudblogs.microsoft.com/quantum/2020/01/21/oti-lumionics-accelerating-materials-design-microsoft-azure-quantum/.

33 https://www.menten.ai/

34 https://www.zapatacomputing.com

35 R. Karp, “Reducibility among Combinatorial Problems.” In: Miller R.E., Thatcher J.W., Bohlinger J.D. (eds) Complexity of Computer Computations. The IBM Research Symposia Series. Springer, Boston, MA (1972).

36 R. Orus, S. Mugel, and E. Lizaso, “Quantum Computing for Finance: Overview and Prospects,” Reviews in Physics 4 (January 2019): 100028 provide an excellent review of possible uses of quantum technology in finance, including references to M.L. de Prado, Generalized optimal trading trajectories: a financial quantum computing application (2015). 10.2139/ssrn.2575184; G. Rosenberg, Finding optimal arbitrage opportunities using a quantum annealer (2016); and A. Milne, M. Rounds, P. Goddard, Optimal feature selection in credit scoring and classification using a quantum annealer (2017).

37 “Optimal Feature Selection in Credit Scoring and Classification Using a Quantum Annealer,” white paper, A. Milne, M. Rounds, and P. Goddard, Vancouver, 2017. http://1qbit.com/wp-content/uploads/2017/04/1QBit_-_Optimal-Feature-Selection-in-Credit-Scoring-and-Classification-Using-a-Quantum-Annealer_-_2017.04.13.pdf.

38 In the quantum context Orus, Mugel, and Lizaso (2019), above footnote 19, reference P. Rebentrost, B. Gupt, and T.R. Bromley, “Quantum Computational Finance: Monte Carlo Pricing of Financial Derivatives,” American Physical Society Physics 98, no. 2 (August 2018) for derivatives and S. Woerner and D.J. Egger, “Quantum Risk Analysis,” npj Quantum Information 5, no. 15 (February 2018): 1-8 for value-at-risk models.

39 A. Mehta, M. Neukirchen, S. Pfetsch, T. Poppensieker, “Managing Market Risk: Today and Tomorrow,” working paper 32, McKinsey and Company, May 2012. https://www.mckinsey.com/~/media/McKinsey/Business%20Functions/Risk/Our%20Insights/Managing%20market%20risk%20Today%20and%20tomorrow/Managing%20market%20risk.ashx.

40 K. Lemme, T.J. Osborne, K.G. Vollbrecht, D. Poulin, and F. Verstraete, “Quantum Metropolis Sampling,” Nature 471, (March 2011): 87–90. M. Szegedy, “Quantum Speed-Up of Markov Chain Based Algorithms” (presentation at the 45th annual IEEE Symposium on the Foundations of Computer Science, Rome, Italy, Oct. 17–19, 2004). A. Montanaro, “Quantum Speedup of Monte Carlo Methods,” Proceedings of the Royal Society A Mathematical Physical and Engineering Sciences 471, no. 2181 (April 2015). C. Ciliberto, M. Herbster, A.D. Ialongo, M. Pontil, A. Rocchetto, S. Severini, and L. Wossnig, “Quantum Machine Learning: A Classical Perspective,” Proceedings of the Royal Society A Mathematical Physical and Engineering Sciences 474, no. 2209 (January 2018).

41 https://www.cogniframe.com

42 https://www.multiversecomputing.com/

43 The insights for this section were generated from discussions with Abhi Rampal, Founder and CEO of Solid State AI.

44 Solidstate.ai works with a variety of machine learning tools to improve the efficiency of advanced manufacturing (https://www.solidstate.ai/).

45 The original DuPont Analysis was developed by an employee at DuPont Corporation in 1912.

46 ROA often adds after tax interest to net income in its numerator. For simplicity in this example, we assume that there is no debt in the firm’s capital structure and thus the numerator simply contains net income.

47 E. Gibney. “The quantum gold rush,” Nature 574, 22 (2019).

48 See, for example, the US Department of Energy’s blueprint for the quantum internet: https://www.energy.gov/sites/prod/files/2020/07/f76/QuantumWkshpRpt20FINAL_Nav_0.pdf.

49 https://www.agnostiqlabs.com/

50 M. Brooks, “Beyond Quantum Supremacy: The Hunt for Useful Quantum Computers,” Nature 574 (2019): 19–21.

